# Water quality assessment in the “German River of the years 2014/2015”: how a case study on the impact of a storm water sedimentation basin displayed impairment of fish health in the Argen River (Southern Germany)

**DOI:** 10.1186/s12302-017-0108-y

**Published:** 2017-03-06

**Authors:** Paul Thellmann, Bertram Kuch, Karl Wurm, Heinz-R. Köhler, Rita Triebskorn

**Affiliations:** 1 Animal Physiological Ecology, University of Tübingen, Auf der Morgenstelle 5, 72076 Tübingen, Germany; 2 Institute for Sanitary Engineering, Water Quality and Solid Waste Management, University of Stuttgart, Bandtäle 2, 70569 Stuttgart, Germany; 3 Gewässerökologisches Labor Starzach, Tulpenstr. 4, 72181 Starzach, Germany; 4 Steinbeis Transfer-Center for Ecotoxicology and Ecophysiology, Blumenstr. 13, 72108 Rottenburg, Germany; 5 Animal Physiological Ecology, Institute for Evolution and Ecology, University of Tübingen, Auf der Morgenstelle 5, Tübingen, 72076 Germany

**Keywords:** Motorway runoff, Sediment toxicity, Histopathology, Fish embryo test

## Abstract

**Background:**

The present work investigates the impact of discharges from a storm water sedimentation basin (SSB) receiving runoff from a connected motorway in southern Germany. The study lasted for almost two years and was aimed at assessing the impact of the SSB on the fauna of the Argen River, which is a tributary of Lake Constance. Two sampling sites were examined up- and downstream of the SSB effluent. A combination of different diagnostic methods (fish embryo test with the zebrafish, histopathology, micronucleus test) was applied to investigate health impairment and genotoxic effects in indigenous fish as well as embryotoxic potentials in surface water and sediment samples of the Argen River, respectively, in samples of the SSB effluent. In addition, sediment samples from the Argen River and tissues of indigenous fish were used for chemical analyses of 33 frequently occurring pollutants by means of gas chromatography. Furthermore, the integrity of the macrozoobenthos community and the fish population were examined at both investigated sampling sites.

**Results:**

The chemical analyses revealed a toxic burden with trace substances (originating from traffic and waste water) in fish and sediments from both sampling sites. Fish embryo tests with native sediment and surface water samples resulted in various embryotoxic effects in exposed zebrafish embryos (Fig. 1). In addition, the health condition of the investigated fish species (e.g., severe alterations in the liver and kidney) provided clear evidence of water contamination at both Argen River sites (Fig. 2). At distinct points in time, some parameters (fish development, kidney and liver histopathology) indicated stronger effects at the sampling site downstream of the SSB effluent than at the upstream site.

**Conclusions:**

Our results clearly showed that the SSB cannot be assigned as the main source of pollutants that are released into the investigated Argen River section. Moreover, we showed that there is moderate background pollution with substances originating from waste waters and traffic which still should be taken seriously, particularly with regard to the impairment of fish health at both investigated field sites. Since the Argen is a tributary of Lake Constance, our results call for a management plan to ensure and improve the river’s ecological stability.

## Background

The European Water Framework Directive (WFD) forms the basis for a sustainable water management policy. With its implementation, the focus was addressed not only to measures like renaturations of stream systems but also to upgrades of wastewater treatment plants with the aim of improving the ecological conditions of streams and reducing discharges of trace pollutants into surface waters. In general, surface waters receive a large number of pollutants in low concentrations [1], especially due to the discharge of diffuse but also point sources like road runoffs in combination with storm water drainage systems. As a consequence, the aquatic fauna frequently experiences chronic exposure to low concentrations of harmful substances, which may result in a serious impairment of the health condition in the affected organisms. When it comes to the question of potential point sources, often little attention is given to storm water relief systems like storm water overflow basins (SOBs), storm water sedimentation basins (SSBs) or road runoff drainage systems. Particularly in the case of heavy rainfall events or enduring snowmelts, these systems gain further importance due to their massive discharge of untreated water (originating from municipal waste waters and road runoff) into the connected surface waters. These discharges contain a mixture of organic and inorganic contaminants that may cause multiple biological effects (e.g., estrogenic, genotoxic, cytotoxic, or carcinogenic effects) due to interactions with multiple target sites in the exposed organisms [2, 3]. Ellis et al. [4] reported that 30% of total hydrocarbons and 50% of the total suspended solids in urban drainage water originate from road runoff. Such runoff contains a complex mixture of pollutants as, e.g., hydrocarbons, dioxins, metals, de-icing salts, and halogenated phenols originating mainly from traffic and vehicle abrasion [5]. These compounds are of particular concern since it has been shown that they can lead to adverse effects in aquatic organisms [6–9]. Once they (especially those with hydrophobic properties) have been emitted, they can accumulate in sediments and biota. One particular problem is that contaminants in sediments rarely cause effects as single substances, but rather in concert with numerous others that are present in complex mixtures [10]. Furthermore, many contaminants accumulate in the sediments of rivers and reach concentrations far higher than those in the surrounding water. As a consequence, benthic organisms such as invertebrates or ground-living fish species run the risk of continuous exposure to both dissolved and sediment-bound contaminants [11].

The present study deals with the investigation of the storm water sedimentation basin (SSB) in Dürren (Allgäu region, Southern Germany), receiving road runoff from a nearby motorway (A 96) during rain fall events. The SSB represents an isolated storm water retention system for the treatment of polluted road runoff during rainfall events and has no connection to the municipal wastewater system. Its purpose is to clarify the incoming road runoff by means of sedimentation of suspended solids and traffic-related pollutants. The upper water fractions are then released by an outflow at one end of the SSB into the Argen River, a tributary of Lake Constance. Notably, heavy rainfall events may thus have a negative impact on the flora and fauna of the receiving stream due to the discharge of large volumes of potentially polluted water. Consequently, the resulting hydraulic shock loads may lead to an acute exposure of aquatic organisms to a complex mixture of traffic-related substances.

The aim of the present study—which has been commissioned by the Regional Council Tübingen—was to investigate whether the discharge from the SSB leads to an impairment of natural resources which are protected by the Flora–Fauna-Habitat (FFH) Directive of the European Union [12]. The Flora–Fauna-Habitat (FFH) Directive or Habitats Directive is a nature conservation directive of the European Parliament which ensures the protection of rare, threatened or endemic species. To investigate the possible impact of the SSB, different methods on different biological levels were chosen. The Regional Council Tübingen demanded investigations on the fish health of protected indigenous fish species like bullhead. Histopathology and the micronucleus test were negotiated as research methods. The assessment of the macrozoobenthos community and chemical analyses with fish tissues were additionally demanded. Therefore, effect-based investigations on fish health, macrozoobenthos integrity, and fish population structure as well as chemical analyses and measurements of physicochemical parameters were conducted within an investigation period of almost 2 years.

## Methods

### Sampling sites and events

In order to investigate the impact of the Dürren storm water sedimentation basin (SSB; 47°43′38.8″N 9°52′22.9″E) on the Argen River, two sampling sites were defined upstream and downstream of its outflow. The sampling site downstream of the SSB effluent (exposure site, code: D) starts immediately at the discharge site of the SSB into the Argen River and ends approximately 200 m downstream of it. The reference site (code: U) starts about 300 m upstream of the SSB outflow and ends approximately 100 m upstream of it.

In total, five sampling events were conducted between April 2013 and October 2014 (Table 1). The water level was in a normal range (mean low water level) at every sampling event, and no heavy rainfall events occurred 14 days prior to the sampling. The absence of heavy rainfall events directly before sampling was of particular importance, since (1) heavy rainfall can lead to a resuspension or remobilization of sediment-bound pollutants (e.g., [13]) (2) the associated floods may result in a displacement of sediments and organisms such as fish (e.g., [14]). A comparison of both sampling sites respectively an investigation on the impact of the SSB directly after a heavy rainfall event would have led to an incorrect assessment. At each sampling event, sediment and surface water samples as well as fish were taken for various analyses in the laboratory. For the investigation of fish health, we caught stationary indigenous fish species such as the European bullhead (*Cottus gobio*) or loach (*Barbatula barbatula*). These benthic fish species have a sedentary behavior, stay close to the river bottom and cover relatively short distances, allowing us to regard the sampling sites up- and downstream of the SSB as being independent from one another. The migration of fish from one sampling site to the other can, therefore, be minimized to the greatest extent. Due to low catching numbers of these two target species in two sampling campaigns, we decided to catch those fish species instead that were most abundant during the respective sampling events (Table 1).

**Table 1:** Conducted sampling events during the investigation period

Sampling event	Code: upstream SSB	Code: downstream SSB	Sampled fish species	Season
1	U1	D1	Rainbow trout (*Oncorhynchus mykiss*)	Spring 2013
2	U2	D2	Loach (*Barbatula barbatula*)	Summer 2013
3	U3	D3	Barbel (*Barbus barbus*)	Autumn 2013
4	U4	D4	Loach (*Barbatula barbatula*)	Spring 2014
5	U5	D5	Bullhead (*Cottus gobio*); Loach (*Barbatula barbatula*)	Autumn 2014

Native sediment and surface water samples were used for the performance of a fish embryo test (FET) with the zebrafish (*Danio rerio*) and, in parallel, for limnological and chemical analyses. Tissues and blood samples from field-caught fish (see Table 1) were used for histopathological investigations and for the micronucleus test, respectively. Furthermore, chemical analyses were conducted with the remaining fish tissues. In parallel, investigations on the macrozoobenthos integrity were conducted using the multi-habitat sampling procedure [15]. These data are presented in the Appendix only but were used for the discussion of the results.

#### Fish sampling

All fish were caught by electrofishing with the permission of the Regional Council of Tübingen (Germany). The assessment of fish stocks was conducted using the length and frequency distribution of caught fishes.

At each of the investigated sampling events, up to ten (*n* > 5) individuals of the same species were retained for biological analyses. Due to the distance between the sampling sites and the rather territorial behavior of the investigated species, it is highly likely that the sampled populations had their respective, distinct exposure history. Blood samples (100–150 µL) for the micronucleus test were immediately collected after the spine cut with a pipette. For histopathological investigations, tissue samples of the gill, liver and kidney were carefully dissected and immediately fixed in 2% glutaraldehyde (Sigma-Aldrich, Germany) dissolved in 0.1 M cacodylate buffer (sodium cacodylate trihydrate, pH 7.6, Sigma-Aldrich, Germany). Fish tissue and blood samples (smeared on microscope slides and fixed in methanol) were stored at 4 °C in a cool box until arrival at the laboratory.

#### Sediment and water sampling

Sediment and surface water samples were taken at each sampling site and at every sampling event. Sediment sampling took place close to the riverside, in which sediment material was taken from the top 2 to 4 cm of the riverbed. To obtain representative sediment samples of each sampling site, sediment samples were taken at 4 to 10 spots within each sampling site and over a sampling distance of 20 to 30 m. After collection, all sediment samples were homogenized with a stainless steel shovel in a stainless steel bucket and divided into batches of 100 or 300 g, wrapped in aluminum foil (Roth, Germany). The 100 g batches were used in the FET with *Danio rerio*, whereas the 300 g batches were used for chemical analyses. Due to financial shortcomings, chemical analyses with sediment samples were only conducted with samples of sampling event 5.

The sampling of surface water was conducted in the main current at a depth of 10–15 cm. All flasks were rinsed with river water before sampling. For the FET with *Danio rerio*, three 250 mL sterilized glass flasks (Schott Duran, Germany) were filled with surface water from each sampling site. Furthermore, effluent samples from the SSB (also in three glass flasks) were taken during every sampling event. These samples were also used for application in the FET. All samples were stored in a cool box at 4 °C during sampling and transport. After arrival at the laboratory, surface water and sediment samples were immediately frozen at −20 °C.

### Physicochemical water parameters

Conductivity, pH, water temperature, and oxygen concentration were measured directly in the stream during every sampling event. The concentrations of nitrate, nitrite, ammonium, (ortho-) phosphate, and chloride were determined photometrically using tube test kits (NANOCOLOR^®^ tube tests) and a compact filter photometer (Compact photometer PF-12Plus, Macherey–Nagel, Düren, Germany). Carbonate and total hardness were determined titrimetrically with test kits (MColortest™, Merck, Darmstadt, Germany). The measured concentrations were assessed using the guidance values defined by the German Act for the Regulation of Surface Waters of 2011 [16] and the German Working Group on Water Issues (LAWA) [17].

In addition, temperature was measured continuously by data loggers (HOBO^®^ Water Temp Pro by Onset Computer Corporation) at both sampling sites and at the SSB effluent. Data loggers were fixed with a metal rod in the riverbed at a depth of 30 cm. To assess the impact of the SSB on the salinity of the Argen River, conductivity and chloride concentration were measured after three rainfall events in March and April 2013. These measurements were conducted at both sampling sites and at the SSB effluent.

### Chemical analyses

Chemical analyses were conducted for 33 compounds including polycyclic aromatic hydrocarbons (PAHs), polychlorinated biphenyls (PCBs), polybrominated diphenyl ethers (PBDEs), polycyclic musk compounds, methyltriclosan, and dichlorodiphenyldichloroethylene (DDE). Detailed information on the analyzed substance groups and substances is provided in Appendix 1 (Table 4).

Two grams of the freeze-dried and homogenized fish or sediment samples was Soxhlet-extracted (100 mL *n*-hexane, 6 h). The extracts were rotavaporated to 10 mL (350 mbar, 40 °C) and stored in 20 mL vials. Aliquots corresponding to 1 g fish sample (dry weight, DW) were transferred into 5 mL vials. After addition of the internal standards (AHTNd^3^: 100 μL, 1 ng/μL AHTN-d^3^ in methanol, 16 perdeuterated PAHs according to US-EPA: 100 μL, DDT- ^13^C_12_, DDE-^13^C_12_, DDD-^13^C_12_: 50 μL, ^13^C_12_-PCB-congeners #28, #52, #101, #118, #138, #153, #180, #194: 50 μL, each 1 ng/L in toluene, Ehrenstorfer GmbH, Augsburg, Germany, LGC Promochem, Wesel, Germany), extracts were concentrated (nitrogen stream, 40 °C) and dissolved in *n*-hexane (200 μL) before complete dryness. The sample was purified via consecutive elution with increasing solvent polarity (*n*-hexane, *n-*hexane/dichloromethane 1:1 v/v, acetone, 5 mL each, LGC Promochem) of the extract on a silica column (1 g). After adding the recovery standard (biphenyl-d10, 100 µL, 1 ng/µL in toluene), the fractions containing the PAHs, DDE and methyltriclosan (*n*-hexane/dichloromethane 1:1 v/v) and the synthetic musks AHTN and HHCB (acetone) were reduced to 50 μL (nitrogen stream, 40 °C).”

Analysis of the sample extracts was performed using gas chromatography (HRGC Agilent 6890N) directly coupled to a mass selective detector (LRMS Agilent 5975N). Automatic injection of 1 μL solution was accomplished in splitless mode at 250 °C. Chromatographic separation was performed on a Varian VF-Xms column (30 m × 0.25 mm × 0.25 μm) under constant flow conditions (helium 5.0, 1 mL/min). The GC oven temperature program was the following: initial temperature 80 °C (held 1 min), 7 °C/min 180 °C (held 1 min), 12 °C/min 240 °C, 20 °C/min 300 °C (held 9 min). Samples were analyzed in single ion monitoring mode using characteristic fragment ions of analytes and corresponding internal standards. The analytes were quantified via the isotope dilution method (PAHs, PCBs, DDE, AHTN) or external calibration with internal reference standards (HHCB, methyltriclosan). Depending on the individual sample matrix, the limit of quantification (LOQ, signal noise ratio 10:1, limit of detection LOD signal noise ratio 3:1) was in the range of 0.17 µg/kg DW (PAHs, PCBs, DDE and methyltriclosan) and 1.7 µg/kg DW (dry weight) for the synthetic musks. LOQs for the individual analytes are listed in Appendix 1 (Table 4). Blank values were determined for the whole analytical procedure (soxhlet-extraction, column clean-up) in triplicate. The blank concentrations which were recalculated to the sample amount used for the extraction are listed in Appendix 1 (Table 4). Blank values were not subtracted from the analytical values. The recovery rates of the isotope-labeled standards were in the range of 88% (dibenzo[ah]antracene-d14) to 97% (^13^C_12_-PCB-153 and AHTN-d^3^). The calculation of the recovery rates is based on the ratio (peak areas) of the individual standard compounds (added to the sample extract prior to the clean up procedure) to the syringe standard. The basis value for 100% recovery was determined by the quantification of a mixture containing the quantification standards and the syringe standard as well in the same ratio as used for the analytical procedure. The recovery rates were not considered in the calculation of the analytical values. Isotope-labeled standard compounds and the reference standards were purchased by Ehrenstorfer GmbH Germany and LGC Standards Germany. Solvents for residual analysis were purchased by Sigma-Aldrich Germany and VWR Germany.

### Biological analyses

#### Fish embryo test

The conducted fish embryo tests generally followed the procedure of the OECD Guideline 236 [18] and were applied and modified as sediment contact assays according to the work of Hollert et al. [19]. For each sampling site and event, three independent test runs (three tests on different dates) were conducted with native surface water and sediment samples, or effluent samples from the SSB, respectively. Thus, one glass flask with surface water and one sediment batch were used for each test run. Oxygen levels were measured at the beginning of the test. Oxygen saturation was always above 90% in the tested surface water samples as well as in the SSB effluent samples. The procedure for the sample preparation and test performance was the same as described in the work of Thellmann et al. [20]. The tests were considered to be valid, when (1) the fertilization rate of all eggs collected was ≥70%, (2) the survival rate of embryos from the negative control was ≥90% after 96 h of exposure (3), and the hatching rate in the negative control was ≥80% after 96 h of exposure. To check the sensitivity of the fish strain, 3,4-dichloroaniline was tested twice a year in a concentration of 4 mg/L according to the OECD Guideline 236. Since an exposure to this concentration resulted in mortality rates greater than 35%, embryos of the used fish strain proved to be suitable for the application in the FET. For each treatment, five glass Petri dishes (30 mm diameter, Schott Duran, Germany) were filled with 2.5 g of the corresponding sediment sample and overlaid with the appropriate surface water from the same sampling site and event. Five additional Petri dishes containing reconstituted water (according to ISO 7346/3) served as negative control. Five fertilized eggs were transferred to each of the used tests dishes, resulting in a total of 25 eggs per treatment and control group. To ensure an optimal and consistent temperature, all test dishes were kept in an incubator at 26 ± 1 °C.

Developmental stages (including developmental delays and failures) as well as mortality rate and hatching rate were observed at defined time points (Appendix 1, Table 5) using binoculars (Stemi 2000-C, Zeiss, Oberkochen, Germany).

#### Histopathology

After fixation in 2% glutardialdehyde for at least one week, tissue samples were dehydrated with ethanol and routinely processed for paraffin embedding. Fish tissue samples (gills, kidney, liver) were embedded in pure paraffin (Carl Roth, Germany) using a tissue processor (Model TP 1020; Leica Biosystems; Germany). Subsequently, each tissue sample was cut into sections of 2 µm thickness using a sliding microtome (SM 2000 R; Leica Biosystems; Germany). Afterwards, one part of the sections was stained with hematoxylin–eosin staining (H&E), enabling the differentiation of cell types and providing an overview of the structure in the observed tissues. The other part of the sections was stained by a periodic acid Schiff reaction, with the aim of providing information on the glycogen or glycoprotein content (e.g., glycogen reserves in the hepatic tissue and mucous cells in the gills) in the observed tissues. The health condition of the tissues was qualitatively described and, in addition, semi-quantitatively assessed according to the method described by Triebskorn et al. [21, 22]. All the investigated histological samples were assessed in an observer-blinded way to prevent observer bias. The semi-quantitative assessment of the overall tissue condition (in the gills, liver, and kidney) was conducted using a five-class ranking system: class 1—control state/no pathological alterations; class 2—slight to moderate alterations; class 3—reaction state/distinct reactions of cells and tissues, slight pathologies; class 4—strong tissue alterations and/or partial necrosis; class 5—destruction state/severe tissue alterations with extended necrotic areas.

#### Micronucleus test

The micronucleus test detects DNA damages, represented by the formation of micronuclei in fish erythrocytes. A high number of micronuclei can be, therefore, regarded as an indicator of genotoxic effects, e.g., [23, 24]. Immediately after blood collection, one drop of fish blood was smeared onto a previously degreased (in 97% ethanol) slide (Langenbrick, Germany) using a cover slip. Two smears were produced for each individual. The slides were subsequently fixed with methanol and stained with Giemsa (Carl Roth, Germany) as described in the work of Rocha et al. [25]. To estimate the total amount of erythrocytes containing micronuclei, 2000 erythrocytes (1000 cells per slide) per individual were counted using a light microscope (Axioskop2; Zeiss, Germany). The scoring and the evaluation of micronuclei were conducted according to the work of Rocha et al. [25]. The number of cells containing micronuclei was expressed as the percentage of the total number of counted erythrocytes.

### Statistical analyses

Statistical analyses were performed using SAS JMP version 11.0 (SAS Institute). Mortality and hatching rates in the FET were analyzed by Cox’s proportional hazards analysis. The developmental failure rate of zebrafish embryos at 96 hpf (hours post-fertilization) was analyzed using Fisher’s exact test. The significance level was set to α = 0.05. To correct for multiple testing, the Holm–Bonferroni method was applied to adjust the significance levels. Data from the micronucleus tests were tested with a paired *t* test. Statistical analysis of the histopathological data was performed using the Likelihood ratio test.

## Results

### Physicochemical water parameters

Overall, both sampling sites revealed a good ecological condition according to the new guidance values defined by LAWA [17] and the 2011 German Regulation Act for Surface Waters [16]. For reasons of simplicity, only the values measured for sampling events 1 and 2 are presented in Table 2. However, the measurements revealed a slightly higher conductivity at the SSB effluent and also a slightly higher conductivity at the exposure site. The values measured for the sampling events 3, 4, and 5 are presented in Appendix 1 (Table 6).

**Table 2:** Physicochemical data of sampling events 1 and 2

	U1	SSB effluent 1	D1	U2	SSB effluent 2	D2
Conductivity (µs/cm)	451	720	540	475	765	509
Water temperature (°C)	13.9**	12.1	13.6**	18.7*	13.6	18.2*
O_2_ saturation (%)	113.5	111	120	116	118.2	111.7
O_2_ content (mg/L)	10.92**	11	11.6**	9.94**	11.31	9.83**
NH_4_–N (mg/L)	0.06*	–	<0.04**	<0.04**	<0.04	<0.04**
NO–N (mg/L)	0.012	–	0.01	0.007	0.01	0.006
NO–N (mg/)L	0.7	–	0.9	0.9	3.8	1.1
PO–P (mg/L)	<0.05*	–	<0.05*	<0.05*	<0.05	<0.05*
Carbonate hardness (°dH)	16	–	15	19	–	19
Overall hardness (°dH)	15	–	16	20	–	20
pH	8.5*	–	8.5*	8.2*	7.95	8.1*
Chloride (mg/L)	11**	–	19**	13**	70	17**

Values were evaluated and assessed according to the guidance values defined by LAWA (German Working Group for Water Issues; LUBW [18]) and the German Regulation Act for Surface Waters of 2011 (OGewV [16])

Values marked with two asterisks (**) point to very good ecological conditions; values marked with one asterisk (*) point to ecological conditions that are at least good

The continuous measurement of the water temperature by data loggers and the conductivity measurements after rainfall events did not show an impact of the SSB on the investigated section at the Argen River. For reasons of simplicity, only the data of four sampling days (with occurring rainfall) are presented in Appendix 2 (Fig. 4).

### Chemical analyses

Chemical analyses were conducted with fish tissues and sediment samples (analyses of sediments only in sampling event 5). Due to financial restrictions, only sediments from sampling event 5 were chemically analyzed. For reasons of simplicity, only the compounds with the highest measured concentrations that are probably responsible for the observed effects in biota are listed in Table 3. The complete table of analyzed compounds can be found in Appendix 1 (Table 7).

**Table 3:** Measured concentrations (in µg/kg, dry weight) of PAHs, PCBs, PBDEs, polycyclic musk compounds, and DDE in fish tissues (pooled fish samples) and sediment samples from the Argen River

Species/sediment	Rainbow trout (*Oncorhynchus mykiss*)		Barbel (*Barbus barbus*)		Loach (*Barbatula barbatula*)		Loach and bullhead (*B. barbatula*; *C. gobio*)		Sediment		EQS in biota [µg/kg] wet weight
Sampling site/compound	U1	D1	U3	D3	U4	D4	U5	D5	U5	D5	
Naphthalene	26.99*	6.26	17.43*	8.97	25.16*	31.78*	10.25	52.70*	2.80	4.30	5
Fluorene	5.10	2.61	2.94	2.25	1.97	2.82	1.28	1.42	2.50	2.40	5
Phenanthrene	16.14	8.75	17.94	12.85	15.37	17.34	12.10	6.94	21.00	28.20	5
Fluoranthene	5.70	3.61	6.14	5.28	5.95	7.84	6.85	5.45	35.40	45.80	30
Pyrene	5.22	3.62	5.56	3.97	5.38	6.97	6.42	4.98	32.10	39.70	5
HHCB	19.14	7.86	25.71	16.74	18.99	13.5	12.9	5.78	4.90	3.70	–
AHTN	6.57	3.96	9.75	8.34	12.43	6.42	6.80	2.38	3.40	2.80	–
DDE	4.30	3.18	3.75	4.14	4.11	3.66	4.83	3.82	0.90	0.80	–
PCB-28	2.33	2.66	1.71	1.59	2.20	1.10	0.90	1.90	0.52	0.61	–
PCB-52	1.53	1.61	0.95	1.31	0.90	1.20	1.10	0.80	0.33	0.28	–
PCB-101	0.90	1.58	0.72	0.45	2.30	2.80	2.40	1.30	0.45	0.51	–
PCB-118	0.90	1.58	0.72	0.45	0.60	0.90	0.50	0.40	0.23	0.22	–
PCB-138	3.99	2.70	3.58	3.21	3.70	4.90	3.50	3.10	0.71	0.62	–
PCB-153	4.49	3.10	3.80	3.55	4.30	5.20	3.70	3.20	0.75	0.68	–
PCB-180	1.09	1.00	0.90	0.78	1.10	1.00	1.20	0.80	0.52	0.56	–
Sum PBDE	1.45*	1.64*	2.37*	1.47*	1.59*	1.55*	4.62*	1.33*	0.69	0.78	0.0085
MTCS	1.24	0.21	1.15	0.64	0.98	1.14	1.51	0.97	1.40	0.90	–

Values marked with an asterisk (*) indicate concentrations that exceeded the environmental quality standards (EQS) of the European Parliament [26]

The assessment of the measured values for PAHs and PBDEs was based on directive 2013/39/EU of the European Parliament [26]. Since benzo(a)pyrene is considered to be a marker for the other PAHs, the environmental quality standard in biota and water for PAHs refers to the concentration of benzo(a)pyrene [26]. Values marked with an asterisk (*) represent concentrations that exceeded the environmental quality standards (EQS) of the European Parliament [26].

All measured concentrations were in the low microgram per kilogram range (dry weight). Fish tissues from sampling events 1 to 5 showed elevated concentrations of PBDEs that exceeded the EQS defined by the European Parliament [26]. With regard to the analyses of PAHs in fish tissues, the highest concentrations were found for naphthalene and phenanthrene. Some of the concentrations measured for naphthalene also exceeded the EQS defined by the European Parliament [26]. The sediments from the exposure site (downstream of the SSB effluent) showed slightly elevated concentrations of some PAHs, PCBs, and PBDEs in comparison to the upstream sediments (Table 3). However, the measured concentrations in fish samples show a contrary picture for some of the analyzed compounds. Here, fish from the upstream (reference) site sometimes showed higher concentrations in comparison to the exposure site. Therefore, a comparison of the measured values (fish tissues and sediments) did neither reveal any clear difference between the investigated sampling sites nor, consequently, any obvious influence of the SSB.

### Biological analyses

#### Fish embryo test

**Fig. 1:**
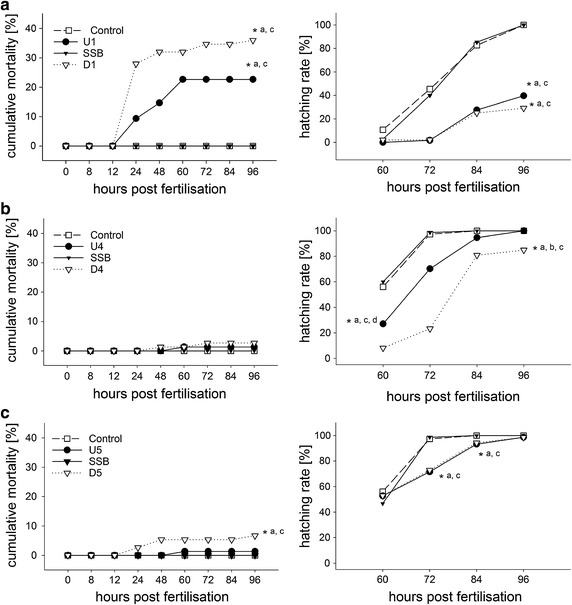
Mortality and hatching rates during the fish embryo test. Zebrafish embryos exposed to water and sediment samples from the Argen River from sampling event 1 (**a**), sampling event 4 (**b**), and sampling event 5 (**c**). **a** Significant differences (*p < 0.05) were found between the sampling sites and the control treatment, respectively the SSB treatment. **b** Significant differences (*p < 0.05) between the sampling sites and the control treatment and also between the sampling sites and the SSB treatment were found for the hatching rate. **c** Significant differences were found between the downstream site and the control treatment, respectively the SSB treatment. The combined data of three test runs were assessed by the Cox proportional hazards survival model

The results of the FET are presented in Fig. 1. Data and standard deviations are presented in Appendix 1 (Table 8).

With regard to the first sampling event, sediment and surface water samples from both sampling sites led to significantly elevated mortality and developmental failure rates and also to a significantly reduced hatching rate. In contrast, water samples from the SSB effluent had no effect on the development of the exposed zebrafish embryos. Surprisingly, all samples (including sediment and surface water samples from both sampling sites) of the sampling events 2 and 3 also had no or only minor effects on zebrafish embryogenesis. In turn, samples from sampling events 4 and 5 again showed stronger effects. Sediment and surface water samples from the exposure site (downstream of the SSB) taken at sampling events 4 and 5 led to significantly reduced hatching rates, elevated mortality rates, and elevated developmental failure rates (e.g., spinal deformation, yolk sac edema; see Appendix 2, Fig. 5) in exposed embryos, whereas fish embryos exposed to samples from the reference site showed no or only slight effects, similarly to the negative control. However, the observed mortality rates in samples from sampling events 4 and 5 were below 10 percent and, therefore, meet the criteria for a valid negative control according to OECD Guideline 236 [18].

#### Histopathology

Histopathological investigations revealed cellular reactions in almost all analyzed fish tissues. Nevertheless, no significant differences could be found between the two investigated sampling sites. Rainbow trout (*Oncorhynchus mykiss*) from the first sampling event showed only slight alterations in the gills, liver, and kidney. The investigated gills displayed partial hyperplasia and hypertrophy of epithelial cells as well as occasional epithelial liftings. In the livers, slight cellular vacuolization and mostly slight depletion of glycogen were observed. Also, smaller parts of the liver showed a slightly increased number of melanomacrophages, indicating an inflammatory response. Alterations in the kidney occurred in the form of vacuolization of the cytoplasm and sporadically present proteinaceous fluids in the lumen. Juvenile barbels (*Barbus barbus*; sampling event 3) revealed a good liver condition with only slight alterations of hepatocytes. However, some individuals showed alterations in the kidney such as strong vacuolizations in the tubule cells, proteinaceous fluids in the lumen and glomeruli with dilated capillaries.

**Fig. 2:**
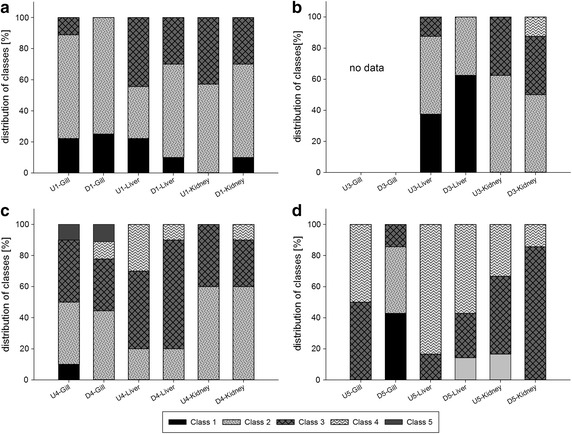
Histopathological classification of gills, liver, and kidney in rainbow trout (**a**), barbel (**b**), loach (**c**), and European bullhead (**d**) from the investigated field sites on the Argen River. Results are presented as the frequency (%) of given evaluation classes for each fish species and organ. Likelihood ratio tests revealed no significant (*p < α = 0.05) differences between the sampling sites

In contrast to the usually slight histopathological alterations in rainbow trout and barbel, the investigated organs of loaches (*Barbatula barbatula*) and bullheads (*Cottus gobio*) sampled during events 2, 4, and 5 revealed severe tissue alterations. The gills showed various reactions in the form of strong hyperplasia and strong hypertrophy of epithelial cells, abundant epithelial liftings, fusion of secondary lamellae, and sporadically occurring necrosis in cells of the secondary lamellae. In the livers, strong vacuolizations in the hepatocytes, strong glycogen depletion, a disintegrated structure of hepatocytes, and irregularly shaped nuclei were observed. On rare occasions, liver tissues revealed lesions in the form of smaller necrotic areas and so-called megalocytes (extremely large hepatocytes, swollen due to an excessive metabolic rate) with irregularly shaped and enlarged nuclei and damaged membranes. Impairments in the kidney occurred in the form of strong vacuolizations in the tubule cells, irregularly shaped nuclei, proteinaceous fluids in the lumen, glomeruli with dilated capillaries, and degenerated glomeruli. The results of sampling events 1, 3, 4, and 5 are shown in Fig. 2.

#### Micronucleus test

Several erythrocytes containing micronuclei could be identified during the investigation of fish blood samples within the micronucleus test. The results of all tests are summarized in the Appendix 2: Fig. 6. A significant variation was found among loaches that were caught at sampling event 4. Here, a higher amount of micronuclei was detected in fish caught downstream. No differences between fish caught up- vs. downstream were observed for the other sampling events.

### Catching numbers of fish species

**Fig. 3:**
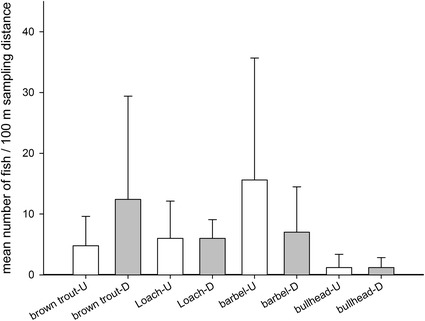
Catching numbers of brown trout, loach, barbel and European bullhead at both of the investigated Argen River sites: *U* upstream, *D* downstream of the SSB effluent. *Vertical bars* include all five sampling events. Fish sampling was conducted over a distance of 200 m

**Fig. 4:**
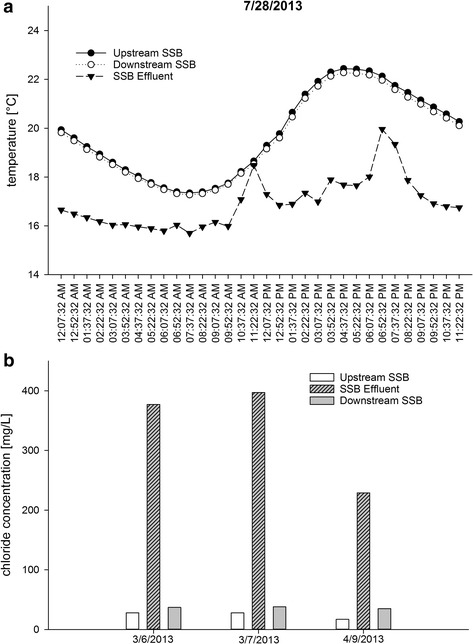
**a** Temporal temperature profile of water temperature at both sampling sites and the SSB effluent during and after a rainfall event on 28 July 2013; **b** measured chloride concentrations at both sampling sites and the SSB effluent after three rainfall events in March and April 2013

**Fig. 5:**
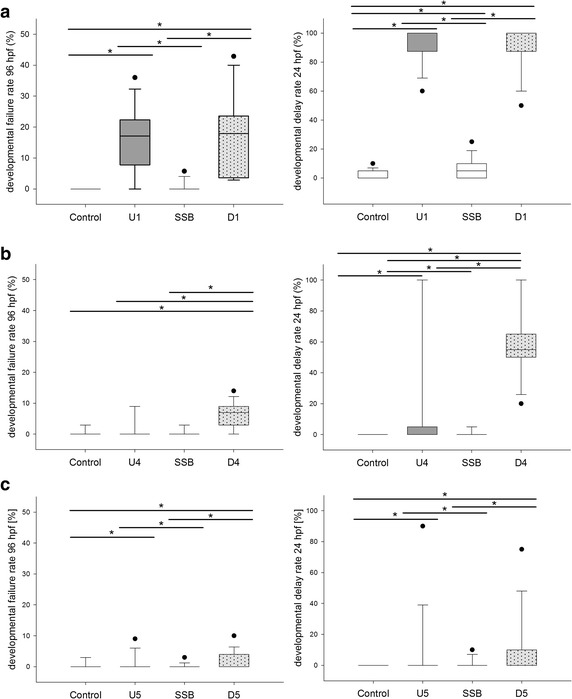
Developmental delay and developmental failure rates observed in the fish embryo test. Zebrafish embryos exposed to water and sediment samples from the Argen River from sampling event 1 (**a**), sampling event 4 (**b**), and sampling event 5 (**c**). Significant differences (*p < 0.05) are indicated with asterisks. The combined data of three test runs were assessed by the Fisher’s exact test. To correct for multiple testing, the Holm–Bonferroni method was applied

**Fig. 6:**
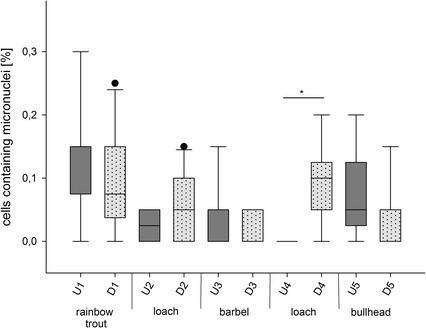
Frequency of erythrocytes containing micronuclei in fish of the Argen River upstream and downstream of the SSB effluent. Results are given as percentages of fish erythrocytes containing micronuclei (total of 2000 counted cells). Data are given as medians, 25th/75th percentiles (*boxes*) and 5th/95th percentiles (*whiskers*). There was a significant difference (*p* < 0.05) between U4 (M = 0; SD ± 0) and D4 (M = 1.78; SD ± 1.2). Analysis of data was conducted using paired t tests

The Argen River represents a typical stream type of the Alpine foreland where brown trout (*Salmo trutta* f. *fario*), European bullhead (*Cottus gobio*), grayling (*Thymallus thymallus*), loach (*Barbatula barbatula*), and barbel (*Barbus barbus*) represent the main indigenous fish species [27]. Almost all typical fish species of this stream type with the exception of lake trout (*Salmo trutta* f. *lacustris*), burbot (*Lota lota*), and the common nase (*Chondrostoma nasus*) were caught at both sampling sites. However, it was conspicuous that the catching numbers of all caught fish species were abnormally low compared to the natural productive capacity of this stream type. From a quantitative point of view, this means that the population structure of all fish species showed considerable deficits. As an example, the catching numbers of loach, barbel, European bullhead, and brown trout are presented in Fig. 3.

## Discussion

Our results indicate background pollution with traffic-related and waste water-related compounds at the investigated field sites of the Argen River.

### Physicochemical parameters

The physicochemical analyses and the temperature measurements by data loggers did not reveal any clear differences between the two investigated sampling sites up- and downstream of the SSB effluent at the Argen River and indicated a good ecological condition according to the guidance values defined by the 2011 German Regulation Act for Surface Waters [16] and the German Working Group on Water Issues (LAWA) [17]. Nevertheless, a slightly higher conductivity was observed at the SSB effluent, which is most likely due to the use of sodium chloride as de-icing agent in winter periods [28]. However, the chloride concentration and the conductivity were only slightly increased at the exposure site and indicated a sufficient dilution by the Argen River. Consequently, there is no clear evidence of an influence of the SSB on the salinity of the Argen River.

### Chemical analyses

The chemical analyses indicated a toxic burden with micropollutants in fish and sediments at both of the investigated sampling sites. With regard to the investigated PAHs, the concentrations for naphthalene exceeded the environmental quality standards (EQS) defined by the European Parliament [26]. The exceedance of the EQS should be alarming since PAHs are known to have genotoxic and carcinogenic effects on fish, lead to morphological abnormalities in fish larvae, and also cause oxidative stress in the organs of exposed fish [7, 29–32]. Besides the aforementioned PAHs, PCBs and PBDEs were also detected in fish from the Argen River, with the measured concentrations for the sum of PBDEs also exceeding the EQS of the European Parliament [26]. PBDEs have a similar structure and similar properties, and may exert similar toxic effects like PCBs, but their C–Br bond makes them more susceptible to environmental degradation than PCBs with their much more stable C–Cl bond [33–35]. Due to their high hydrophobicity, both substance groups tend to accumulate and biomagnify in organisms where they can reach concentrations that are far higher than those in water [36, 37]. Especially problematic is the metabolic debromination of higher brominated PBDE congeners, as reported by Roberts et al. [38]. Taking this into account, the concentrations of lower brominated PBDEs, and thus presumably also their toxicity, may be enhanced due to the metabolic debromination of higher brominated congeners, as shown by Tomy et al. [39]. Investigations of Brown and Peake [40] showed that road debris represents the main source of PAHs in road runoff and storm water discharges. Benfenati et al. [41] reported that traffic represents the main source of roadside pollution with PAHs, PCBs, and other organic pollutants. Hence, in this case the analyzed substances like PAHs and PCBs are strongly supposed to originate from the road traffic near the Argen River.

Predatory fish or birds with a long life span located at the top of the food web can concentrate high amounts of harmful hydrophobic substances like PAHs [2, 42]. Thus, a frequent uptake of, for example, PAHs over a long period could lead to severe health impairments in aquatic organisms, especially in combination with other harmful substances like PCBs and PBDEs. In addition to that, Lema et al. [43] assumed that PBDEs may be transferred maternally into the lipid stores of oocytes, and thus the offspring could be exposed to PBDEs during embryogenesis. Another non-negligible problem is the bioaccumulation of hydrophobic substances in riverine sediments. PAHs are usually attached to particles and organic matter [44], by which sediments can act as both sinks and secondary sources for hydrophobic compounds [45]. Due to the remobilization via bioturbation or flood events [46, 47], sediment-bound pollutants can again become available for aquatic organisms and consequently affect their health [48]. Indigenous fish species like brown trout (*Salmo trutta* f. *fario*) and grayling (*Thymallus thymallus*) lay their eggs on those parts of the riverbed that are characterized by gravel and finer sediment particles and can, therefore, be negatively influenced by contaminated sediments.

In summary, it seems very likely that the analyzed substances are not only responsible for the observed reactions in the investigated fish tissues and the fish embryo tests, but also for the low density of all caught fish species.

The synthetic musks HHCB (galaxolide) and AHTN (tonalide) and the triclosan derivative MTCS (methyltriclosan) represent compounds that originate from personal care products and are, therefore, indicators of background pollution with municipal waste waters. For HHCB, it was demonstrated that it can cause anti-estrogenic effects in in vivo and also in vitro test systems [49, 50]. Luckenbach et al. [51] showed that both AHTN and HHCB inhibit the multixenobiotic defense system in the California mussel (*Mytilus californianus*). AHTN also affected the heart rate in exposed zebrafish embryos (*Danio rerio*) as shown by Carlsson and Norrgren [52].

### Biological data

The conducted fish embryo tests resulted in varying embryotoxic effects, depending on the sampling event and site. In samples from sampling event 1, both sampling sites had distinct negative effects on the survival rate, the hatching rate, and the developmental failure rate of exposed zebrafish embryos. One explanation for these effects might be the loading of the sediments with pollutants like PAHs, PCBs, and PBDEs. Experiments by Cachot et al. [53] and McElroy et al. [54] indicated that PAHs can pass the chorion when fish embryos are exposed to benzo[a]pyrene-spiked sediments. As a consequence, sediments containing large amounts of hydrophobic substances may affect the embryonic development of fish embryos. Additionally, it has to be mentioned that the FET with native sediment samples characterizes the overall quality of the sample including both micropollutants but also sediment characteristics as, e.g., particle size or its loading with organic compounds that may lead to oxygen depletion. In contrast to the results of sampling event 1, samples from sampling events 2 and 3 only caused weak effects such as developmental delays in some of the exposed larvae. One probable explanation for this variation is the historic and severe flood event that affected central Europe (including the Argen River) in June 2013, prior to sampling events 2 and 3. The prevalent hydraulic forces may have led to a runoff or shift of the sediments and their bound pollutants and, therefore, may have resulted in distinct lower effects in the FET with samples taken at the events 2 and 3.

Fish embryo tests with samples from sampling events 4 and 5 again revealed stronger effects in zebrafish embryos that were exposed to sediment and surface water samples from the downstream exposure site, indicating a slightly negative influence of the SSB, eventually due to a discharge of traffic-related pollutants into the Argen River. However, the observed mortality rates were below 10% and, therefore, meet the criteria for a valid negative control according to OECD Guideline 236 [18]. Hence, it is unclear whether these results really reflect an adverse effect of the tested sediment samples taken at the sampling events 4 and 5. The chemical analyses showed that the investigated sediments contained PAHs, PCBs, and PBDEs, although in lower concentrations than in the investigated fish tissues. Perrichon et al. [8] exposed zebrafish and Japanese medaka (*Oryzias latipes*) embryos to artificial sediments spiked with benzo[a]pyrene and fluoranthene. The authors found that benzo[a]pyrene caused delayed hatching in exposed medaka embryos, whereas fluoranthene led to increased mortality rates, a lack of hatching, and alterations in growth and development in exposed zebrafish embryos. Additionally, Usenko et al. [55] showed that PBDEs cause developmental malformations and increase the mortality of exposed zebrafish embryos. Although all concentrations were much lower in the examined sediments of our study, one has to take into account that indigenous fish species have much longer developmental periods and, therefore, spend more time lying on the riverbed during their embryogenesis. As a consequence, they experience a much longer exposure time than zebrafish or medaka embryos in laboratory studies. Hence, the observed loading with pollutants of the Argen sediments could also be an explanation for the low fish densities due to an impairment of embryogenesis in the affected fish embryos.

The most obvious effects were shown by the histopathological investigations. Here, fish from both sites, up- and downstream of the SSB, revealed strong reactions or severe health impairments in the examined tissues. The observed effects represent cellular reactions, which can be caused by a great variety of chemical stressors (e.g., heavy metals, organic compounds, salts) and may consequently result in a strong impairment or even loss of function in the affected organs [21]. The gills of the examined fish revealed hyperplasia and hypertrophy of epithelial cells, epithelial liftings, fusion of secondary lamellae, and sporadically occurring necrosis in cells of the secondary lamellae. The observed reactions may result in a reduction of oxygen uptake into the organism and thus negatively affect its metabolism. Santos et al. [9] observed similar reactions in the gills of juvenile Florida pompano (*Trachinotus carolinus*) after a chronic exposure to naphthalene. Barja-Fernández et al. [56] exposed turbot (*Psetta maxima*) to BDE-47 in the lower µg/L range and observed fusions of the secondary lamellae as well as hyperplasia and hypertrophy of the epithelial cells. Shao et al. [57] exposed rainbow trout gill cells (RTgill-W1) to BDE-47 and observed a loss of the cell viability in this cell type. In liver, we observed vacuolizations, a depletion of the glycogen content, an increased number of melanomacrophages, a disintegrated structure of hepatocytes, cellular hypertrophy, and irregularly shaped nuclei. These reactions indicate an increased metabolic rate, which in turn can be an indicator of the metabolic degradation of harmful substances like, for example, PBDEs and PAHs. The livers of turbot (*Psetta maxima*) showed similar reactions (irregular morphology of hepatocytes, cellular and nuclear hypertrophy) after exposure to BDE-47 as demonstrated in the work of Barja-Fernández et al. [56]. A comparison of our results with the literature shows that the analyzed substances like PAHs and PBDEs probably contributed to the pathologic alterations in the investigated fish tissues. However, a clear link between the observed effects and the analyzed substances cannot be established, since the chemical analyses focused on substances which mainly represent priority substances. The presented chemical data only give an overview on the general status of pollution in the investigated Argen River section. It can be assumed that, in addition, other unidentified pollutants acted as additional stressors and contributed to the observed effects.

The micronucleus test with blood samples of fish from the Argen River revealed micronuclei in almost all the investigated samples. With regard to the investigated blood samples from rainbow trout, a comparison with the literature [58] showed that the amount of micronuclei lies within the range of spontaneously induced micronuclei and can be, therefore, classified as nonhazardous. Due to the lack of reference values for loach (*Barbatula barbatula*) and barbel (*Barbus barbus*), a classification of the measured values proved to be difficult. Boettcher et al. [59] examined barbel from the Danube River between the cities of Sigmaringen and Ehingen, with barbel from an almost nonpolluted site at Sigmaringen showing micronucleus frequencies of 0.14% which surpass those of the juvenile barbel that were caught at the investigated field sites on the Argen River. A comparison with our data indicates that a genotoxic effect on barbel at the investigated sampling sites can, therefore, be excluded.

**Fig. 7:**
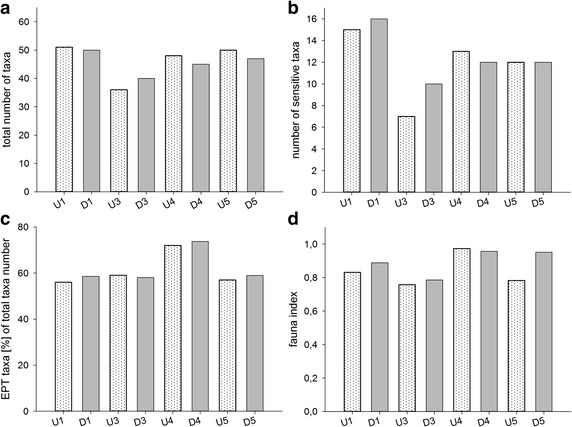
Macrozoobenthos indices: **a** total number of all observed taxa; **b** number of sensitive taxa; **c** EPT (Ephemeroptera, Plecoptera and Trichoptera) taxa; **d** German Fauna Index

The quality assessment of the macrozoobenthos community (see Appendix 2: Fig. 7) by means of the Saprobic Index revealed only a low anthropogenic burden at both investigated field sites. The Saprobic Index, the EPT Taxa, the Rheoindex, and the German Fauna Index point to a good ecological condition of the macrozoobenthos community at both field sites. The amount of caught fish species mostly complies with the natural reference fish fauna of this stream type [27]. Due to the hydraulic and morphologic characteristics of this stream type and the naturally low abundance of food sources (e.g., gammarids), the Argen River has a naturally low fish stock in comparison to other rivers like the Danube or the Neckar. However, from a quantitative point of view, all fish species showed significant deficits at both sampling sites. For example, brown trout (*Salmo trutta* f. *fario*), which normally represents the dominant fish species, was only caught in very low numbers. A high ecological pressure by fishing activities which could have contributed to the low fish numbers can be excluded, since the investigated sections are privately owned and fishing is rarely conducted. Also, a lack of breeding grounds can be excluded, since shallow gravel banks exist at both sampling sites. One probable reason for the low numbers of fish may be predation by black cormorants (*Phalacrocorax carbo*) which is also reflected by the low catching numbers of fish sized between 15 and 40 cm. However, this does not fully explain the low numbers of caught bullhead and loach. It is documented that high predatory pressure by cormorants can lead to a mass development of small fish species like bullhead, loach or minnow [60], which obviously was not the case at the investigated Argen River sections. Moreover, almost all examined fish tissues from loach (*Barbatula barbatula*) and bullhead (*Cottus gobio*) showed strong cellular reactions which are rather caused by pollution than by predatory stress. The overall result is that the documented fish population remains below the natural productive capacity of the Argen River. The shortage of food sources (e.g., invertebrates) can be excluded as a possible reason for the small number of caught fish (in comparison to the natural reference fish fauna of this stream type), since the macrozoobenthos community was in a good condition. Hence, there must be other reasons, such as pollution with, e.g., hydrophobic contaminants by diffuse sources (e.g., roads, traffic, and agricultural land use) or point sources upstream of the reference site. Overall, in terms of the qualitative and quantitative composition of the macrozoobenthos community and the fish densities, there was no recognizable impairment due to the discharge from the SSB.

Summarizing the above, our results demonstrated a serious impairment of fish health, a loading with embryotoxic potentials in the examined Argen River sediments, and also low fish densities at both of the investigated sampling sites. Moreover, the chemical analyses revealed a burden with mainly traffic-related and waste water-related compounds, e.g., PAHs, PCBs, PBDEs, AHTN and MCTS in fish and sediments from both investigated sampling sites. Chemical analyses were mainly focused on compounds which are identified as priority substances. These data only give an overview on the general status of pollution at the respective sampling sites. In the literature, it is documented that priority pollutants may only count for a minor part of the biological response (e.g., [61, 62]). Consequently, it is likely that other pollutants could have contributed to the observed effects or even have acted as the main causal agents. The histopathological investigations demonstrated that sediment-living fish species like the European bullhead (*Cottus gobio*) and loach (*Barbatula barbatula*) reacted much more strongly than pelagic fish species, e.g., rainbow trout (*Oncorhynchus mykiss*), indicating that one of the causal agents may be found within the sediment of the Argen River. This assumption is supported by the results of the fish embryo tests (Fig. 1). The hazardous potential of the sediment can likely be attributed to a burden with lipophilic chemical compounds (Table 3), which obviously originate from traffic and waste waters.

It remains to be noted that in the present case, temporary discharges—not only from the SSB but also from diffuse point sources like runoff from agricultural areas—are of special importance for the condition of the investigated Argen River section. With regard to the biological relevance of the observed effects, it must be mentioned that the Argen River is one of the biggest tributaries of Lake Constance. Lake Constance is not only of economic and environmental importance, but it also serves as a drinking water reservoir for all neighboring states. Hence, the condition of the Argen River has to be investigated over its complete length, in order to ensure its ecological status but also the ecological status of Lake Constance.

In conclusion, it should be noted that—on the basis of the present results—the SSB has no negative impact on the investigated Argen River section.

## Conclusions

In summary, the following insights were gained:

1. The conducted analyses revealed an impairment of fish health and fish development as well as a toxic burden with trace substances at both sampling sites. It was shown that the investigated sediments pose a toxic risk not only for fish embryos but also for benthic living fish. Due to the shortage of dose–response studies for many of the analyzed substances, it cannot be unambiguously clarified in this study which of the substances finally caused or contributed to the observed effects in the investigated fish tissues, blood samples, and fish embryos. Consequently, further investigations are urgently needed. These investigations should also include effect-directed analyses to detect non-target pollutants.
2. The results indicate that the SSB cannot be assigned as the main source of pollutants released into the Argen River, due to a background pollution level at both investigated sites. However, an additional impairment of aquatic organisms due to the discharge of pollutants, e.g., PAHs or metals, cannot be clearly excluded, especially in the case of local heavy rainfall events in combination with a low water level in the Argen River.
3. The Argen River was nominated for the title “River of the years 2014 and 2015”. This nomination may give rise to the impression that the Argen River represents a rather unimpaired surface water ecosystem. The results obtained in our study clearly demonstrate that this is certainly not the case. Furthermore, they showed that there is a moderate background pollution level comprising substances originating from waste waters and traffic at the investigated field sites. As the Argen is considered to be a river of both high ecological and economic importance, our results call for a future monitoring program to broaden the data basis and, in the long run, eventually even for a management plan to ensure and improve the river’s ecological stability. This is also of great importance for the quality and ecological stability of Lake Constance, since the Argen River flows into Lake Constance.

## Appendix

### Appendix 1

See Tables 4, 5, 6, 7 and 8.

**Table 4:** Analyzed substance groups from chemical analyses

	LOQ in µg/kg DW	Blank (whole method) in µg/kg DW
Polycyclic aromatic hydrocarbons (PAHs)		
Naphthalene	0.15	0.15
Acenaphthene	0.02	<LOQ
Acenaphthylene	0.02	<LOQ
Fluorene	0.03	<LOQ
Phenanthrene	0.07	0.07
Anthracene	0.03	<LOQ
Fluoranthene	0.08	0.08
Pyrene	0.08	0.08
Benz[a]anthracene	0.03	<LOQ
Chrysene	0.03	<LOQ
Benzo[b]fluoranthene	0.05	<LOQ
Benzo[k]fluoranthene	0.05	<LOQ
Benzo[a]pyrene	0.10	<LOQ
Indeno[123-d]pyrene	0.12	<LOQ
Benzo[ghi]perylene	0.14	<LOQ
Dibenzo[ah]anthracene	0.17	<LOQ
Polycyclic musks; other compounds		
Galaxolide (HHCB)	1.7	<LOQ
Tonalide (AHTN)	0.6	<LOQ
Methyltriclosan (MTCS)	0.08	<LOQ
Dichlorodiphenyl-dichloroethylene	0.07	<LOQ
Polychlorinated biphenyls (PCBs)		
PCB-28	0.02	<LOQ
PCB-52	0.02	<LOQ
PCB-101	0.03	<LOQ
PCB-118	0.03	<LOQ
PCB-153	0.04	<LOQ
PCB-138	0.04	<LOQ
PCB-180	0.10	<LOQ
Polybrominated diphenyl ethers (PBDEs)		
BDE-28	0.02	<LOQ
BDE-47	0.02	<LOQ
BDE-99	0.03	<LOQ
BDE-100	0.03	<LOQ
BDE-153	0.05	<LOQ
BDE-154	0.05	<LOQ

**Table 5:** Observed developmental stages and endpoints during fish embryo test with *Danio rerio;* indicators of lethality are marked with an asterisk (*)

Endpoints	Hours post-fertilization (hpf)							
	8 h	12 h	24 h	48 h	60 h	72 h	84 h	96 h
Mortality/coagulation*	X	X	X	X	X	X	X	X
Hatching					X	X	X	X
Developmental retardations								
Epiboly	X							
Gastrulation		X						
Formation of somites*			X					
Tail detachment*			X					
Spontaneous movements			X					
Eye development			X					
Heart beat rate (beats/min)*				X				
Otolith formation					X			
Occurrence of melanocytes					X			
Developmental failures								
Edema (heart and yolk)					X			
Malformation of eyes					X	X	X	X
Spinal deformation (scoliosis)					X	X	X	X
Pigmentation failures					X	X	X	X

**Table 6:** Physicochemical data of sampling events 1 and 2

	U3	SSB effluent 3	D3	U4	SSB effluent 4	D4	U5	SSB effluent 5	D5
Conductivity (µs/cm)	460	605	474	476	950	490	506	756	520
Water temp. (°C)	10.5**	10.4	9.8**	15.7**	13	14.8**	12.4**	13.2	12.4**
O_2_ saturation (%)	100	91.1	96	107.0	103	110	102.4	95.1	97.7
O_2_ content (mg/L)	10.41**	9.47	10.1**	9.9**	10	10.4**	10.27**	9.28	9.66**
NH–N (mg/L)	<0.04**	0.07	<0.04**	<0.04**	0.09	<0.04**	<0.04**	<0.04	<0.04**
NO–N (mg/L)	0.005	0.016	0.006	0.01	0.015	0.01	0.005	0.01	0.005
NO_3_–N (mg/L)	0.7	2	0.9	1.10	2.8	1	1.2	3.9	1.1
PO_4_-P (mg/L)	<0.05*	<0.05	<0.005*	<0.005*	<0.05	<0.005*	<0.005*	<0.05	<0.005*
Carbonate hardness (°dH)	18	21	17	16.00	20	16	16	21	17
Overall hardness (°dH)	18	21	18	17.00	22	18	19	23	18
pH	8.27*	8.1	8.23*	8.40*	8.25	8.4*	8.4*	8.25	8.4*
Chloride (mg/L)	8**	61	11**	11.00	121	13*	11*	42	15*

Values were evaluated and assessed according to the new guidance values defined by LAWA (German Working Group for Water Issues; LUBW [17]) and the German Regulation Act for Surface Waters of 2011 (OGewV [16])

Values marked with two asterisks (**) point to very good ecological conditions; values marked with one asterisk (*) point to ecological conditions that are at least good

**Table 7:** Measured concentrations (in µg/kg, dry weight) of PAHs, PCBs, PBDEs, polycyclic musk compounds, DDE and MTCS in fish tissues and sediment samples of the Argen River

Species/sediment	Rainbow trout (*Oncorhynchus mykiss*)		Barbel (*Barbus barbus*)		Loach (*Barbatula barbatula*)		Loach and bullhead (*Barbatula barbatula*; Cottus gobio)		Sediment	
Sampling site/compound	U1	D1	U3	D3	U4	D4	U5	D5	U5	D5
Naphthalene	26.99*	6.26	17.43	8.97	25.16*	31.78*	10.25	52.70*	2.80	4.30
Acenaphthene	0.83	0.68	0.93	1.12	1.35	1.98	1.07	1.25	1.40	2.10
ACENAPHTHYLENE	1.09	0.64	0.68	0.97	0.88	1.24	0.54	0.66	1.10	1.00
Fluorene	5.10	2.61	2.94	2.25	1.97	2.82	1.28	1.42	2.50	2.40
Phenanthrene	16.14	8.75	17.94	12.85	15.37	17.34	12.10	6.94	21.00	28.20
Anthracene	1.67	0.88	2.45	1.97	1.55	4.21	1.35	1.21	3.50	6.40
Fluoranthene	5.70	3.61	6.14	5.28	5.95	7.84	6.85	5.45	35.40	45.80
Pyrene	5.22	3.62	5.56	3.97	5.38	6.97	6.42	4.98	32.10	39.70
Benz[a]-anthracene	0.50	0.44	0.53	0.48	0.61	0.71	0.97	0.65	16.30	14.70
Chrysene	0.46	0.43	0.41	0.37	0.52	0.68	1.52	0.50	18.90	20.30
Benzo[b]-fluoranthene	0.40	0.33	0.38	0.31	0.32	0.28	0.42	0.39	15.4	21.9
Benzo[k]-fluoranthene	0.33	0.26	0.29	0.27	0.29	0.31	0.34	0.38	10.1	16.2
Benzo[a]-pyrene	0.32	0.23	0.24	0.26	0.24	0.21	0.19	0.18	10.8	15.5
Indeno[123-cd]-pyrene	0.14	0.14	0.14	0.11	0.13	0.15	0.14	0.21	4.50	5.80
Benzo[ghi]-perylene	0.16	0.17	0.17	0.15	0.16	0.21	0.19	0.25	5.90	7.50
Dibenzo[ah]-anthracene	0.05	0.05	0.05	0.05	0.05	0.05	0.05	0.05	2.70	2.50
HHCB	19.14	7.86	25.71	16.74	18.99	13.5	12.9	5.78	4.90	3.70
AHTN	6.57	3.96	9.75	8.34	12.43	6.42	6.80	2.38	3.40	2.80
MTCS	1.24	0.21	1.15	0.64	0.98	1.14	1.51	0.97	1.40	0.90
DDE	4.30	3.18	3.75	4.14	4.11	3.66	4.83	3.82	0.90	0.80
PCB-28	2.33	2.66	1.71	1.59	2.20	1.10	0.90	1.90	0.52	0.61
PCB-52	1.53	1.61	0.95	1.31	0.90	1.20	1.10	0.80	0.33	0.28
PCB-101	0.90	1.58	0.72	0.45	2.30	2.80	2.40	1.30	0.45	0.51
PCB-118	0.90	1.58	0.72	0.45	0.60	0.90	0.50	0.40	0.23	0.22
PCB-138	3.99	2.70	3.58	3.21	3.70	4.90	3.50	3.10	0.71	0.62
PCB-153	4.49	3.10	3.80	3.55	4.30	5.20	3.70	3.20	0.75	0.68
PCB-180	1.09	1.00	0.90	0.78	1.10	1.00	1.20	0.80	0.52	0.56
Sum PBDE	1.45*	1.64*	2.37*	1.47*	1.59*	1.55*	4.62*	1.33*	0.69	0.78

Values marked with an asterisk (*) indicate concentrations that exceeded the environmental quality standards of the European Parliament [26]

**Table 8:** Mortality and hatching rates during fish embryo test

	Control		U1		SSB1		D1	
	Mean	SD	Mean	SD	Mean	SD	Mean	SD
Cumulative mortality (%)								
0 hpf	0.00	0.00	0.00	0.00	0.00	0.00	0.00	0.00
8 hpf	0.00	0.00	0.00	0.00	0.00	0.00	0.00	0.00
12 hpf	0.00	0.00	0.00	0.00	0.00	0.00	0.00	0.00
24 hpf	0.00	0.00	9.33	6.11	0.00	0.00	28.00	14.42
48 hpf	0.00	0.00	14.67	16.17	0.00	0.00	32.00	14.42
60 hpf	0.00	0.00	22.67	16.65	0.00	0.00	32.00	14.42
72 hpf	0.00	0.00	22.67	16.65	0.00	0.00	34.67	16.65
84 hpf	0.00	0.00	22.67	16.65	0.00	0.00	34.67	16.65
96 hpf	0.00	0.00	22.67	16.65	0.00	0.00	36.00	18.33
Hatching rate (%)								
60 hpf	10.67	2.31	0.00	0.00	2.67	4.62	2.04	2.75
72 hpf	45.33	15.14	1.72	2.41	40.00	30.20	2.04	2.75
84 hpf	82.67	19.73	27.59	28.13	85.33	27.71	25.00	17.64
96 hpf	100.00	0.00	39.66	28.69	100.00	0.00	29.17	21.64

	Control		U4		SSB4		D4	
	Mean	SD	Mean	SD	Mean	SD	Mean	SD
Cumulative mortality (%)								
0 hpf	0.00	0.00	0.00	0.00	0.00	0.00	0.00	0.00
8 hpf	0.00	0.00	0.00	0.00	0.00	0.00	0.00	0.00
12 hpf	0.00	0.00	0.00	0.00	0.00	0.00	0.00	0.00
24 hpf	0.00	0.00	0.00	0.00	0.00	0.00	0.00	0.00
48 hpf	0.00	0.00	0.00	0.00	0.00	0.00	1.33	2.31
60 hpf	0.00	0.00	1.33	2.31	0.00	0.00	1.33	2.31
72 hpf	0.00	0.00	1.33	2.31	0.00	0.00	2.67	2.31
84 hpf	0.00	0.00	1.33	2.31	0.00	0.00	2.67	2.31
96 hpf	0.00	0.00	1.33	2.31	0.00	0.00	2.67	2.31
Hatching rate (%)								
60 hpf	56.00	4.00	27.03	6.27	60.00	6.93	8.22	13.86
72 hpf	97.33	2.31	70.27	26.46	98.67	2.31	23.29	14.81
84 hpf	100.00	0.00	94.59	9.24	100.00	0.00	80.82	14.72
96 hpf	100.00	0.00	100.00	0.00	100.00	0.00	84.93	12.80

	Control		U5		SSB5		D5	
	Mean	SD	Mean	SD	Mean	SD	Mean	SD
Cumulative mortality (%)								
0 hpf	0.00	0.00	0.00	0.00	0.00	0.00	0.00	0.00
8 hpf	0.00	0.00	0.00	0.00	0.00	0.00	0.00	0.00
12 hpf	0.00	0.00	0.00	0.00	0.00	0.00	0.00	0.00
24 hpf	0.00	0.00	0.00	0.00	0.00	0.00	2.67	4.62
48 hpf	0.00	0.00	0.00	0.00	0.00	0.00	5.33	9.24
60 hpf	0.00	0.00	1.33	2.31	0.00	0.00	5.33	9.24
72 hpf	0.00	0.00	1.33	2.31	0.00	0.00	5.33	9.24
84 hpf	0.00	0.00	1.33	2.31	0.00	0.00	5.33	9.24
96 hpf	0.00	0.00	1.33	2.31	0.00	0.00	6.67	11.55
Hatching rate (%)								
60 hpf	56.00	4.00	52.70	26.21	46.67	6.11	52.86	42.02
72 hpf	97.33	2.31	71.62	24.17	98.67	2.31	72.86	49.49
84 hpf	100.00	0.00	93.24	6.33	100.00	0.00	94.29	11.00
96 hpf	100.00	0.00	98.65	2.41	100.00	0.00	98.57	0.00

Data are presented as mean values (=mean) and standard deviations (=SD)

*hpf* hours post-fertilization

### Appendix 2

See Figs. 4, 5, 6 and 7.

## Abbreviations

EQS: environmental quality standards
SSB: storm water sedimentation basin
WFD: water framework directive
FFH directive: Flora–Fauna-Habitat Directive
FET: fish embryo test
LAWA: German Working Group on Water Issues
PAHs: polycyclic aromatic hydrocarbons
PCBs: polychlorinated biphenyls
PBDEs: polybrominated diphenyl ethers
DDE: 1,1-*bis*-(4-chlorophenyl)-2,2-dichloroethene
MTCS: methyltriclosan
AHTN: tonalide (6-Acetyl-1,1,2,4,4,7-hexamethyltetralin)
HHCB: galaxolide (1,3,4,6,7,8-Hexahydro-4,6,6,7,8,8-hexamethylcyclopenta(g)-2-benzopyran)
